# Prevalence of metabolic syndrome among patients with multiple sclerosis: a systematic review and meta-analysis

**DOI:** 10.1097/MS9.0000000000005633

**Published:** 2026-10-27

**Authors:** Shahriar Ghodous, Saisree Reddy Adla Jala, Kavya Priya Somu, Nikitha Chellapuram, Artika Misra, Abinash Mahapatro, Mohammad Amouzadeh-Lichahi, Maryam Jafari, Amir Khanmirzaei, Mohammad Sammak Salekdeh, Arman Habibi, Nazila Sattari, Negin Letafatkar, Ehsan Amini-Salehi

**Affiliations:** a Gastrointestinal and Liver Diseases Research Center, Guilan University of Medical Sciences, Rasht, Iran; b Mission Hospital, Asheville, NC, USA; c Centinela Hospital Medical Center 555 E Hardy St, Inglewood, CA, USA; d Cooper University Hospital, Cooper Plaza, Camden, NJ, USA; e Mayo Clinic Health System, Mankato, MN, USA; f SIU School of Medicine, Springfield, IL, USA; g Student Research Committee, School of Medicine, Anzali International Campus, Guilan University of Medical Sciences, Rasht, Iran; h Faculty of Medicine, Shahroud University of Medical Sciences, Shahroud, Iran

**Keywords:** meta-analysis, metabolic syndrome, multiple sclerosis, prevalence

## Abstract

**Background:**

Metabolic syndrome (MetS) is a common metabolic condition that coexists with multiple sclerosis (MS), yet reported prevalence estimates vary across studies and populations. This study aims to report the prevalence of MetS among patients with MS.

**Methods:**

Observational studies reporting the prevalence of MetS among patients with MS were included. Pooled prevalence estimates were calculated using random-effects models.

**Results:**

The pooled prevalence of MetS among patients with MS was 25% (95% CI, 20–31%), with substantial heterogeneity (*I*^2^ = 71.87%). In subgroup analyses by MS diagnostic criteria, studies using the McDonald criteria reported a pooled prevalence of 29% (95% CI, 24–34%), and the study using International Classification of Primary Care, Second Edition (ICPC-2)/International Classification of Diseases, Ninth Revision (ICD-9) criteria reported 31% (95% CI, 25–37%). By MetS diagnostic criteria, pooled prevalence estimates were 30% (95% CI, 26–34%) using National Cholesterol Education Program Adult Treatment Panel III, 23% (95% CI, 17–31%) using International Diabetes Federation, and 16% (95% CI, 11–21%) using American Heart Association/National Heart, Lung, and Blood Institute criteria. By body mass index (BMI) category, pooled prevalence was 11% (95% CI, 2–26%) for BMI < 25, 31% (95% CI, 21–42%) for 25 ≤ BMI < 30, and 58% (95% CI, 42–73%) for BMI ≥ 30. By expanded disability status scale (EDSS) category, pooled prevalence was 28% (95% CI, 21–34%) for EDSS < 4 and 51% (95% CI, 19–83%) for EDSS ≥ 4.

**Conclusion:**

MetS is present in approximately one-quarter of patients with MS, although the precision of the estimate is limited by the small number of studies and substantial heterogeneity. Differences in study design, population characteristics, and diagnostic criteria may contribute to this variability. Further studies using standardized definitions and consistent reporting are needed to improve the comparability of prevalence estimates across settings.

## Introduction

Multiple sclerosis (MS) is a chronic, immune-mediated inflammatory disease of the central nervous system and a leading cause of non-traumatic neurological disability among young and middle-aged adults worldwide^[^1,2^]^. Although MS has traditionally been viewed as a primarily neurological disorder, growing evidence indicates that it is frequently accompanied by systemic comorbidities, particularly metabolic and cardiovascular conditions^[^3,4^]^. These comorbidities may substantially influence disease progression, disability accumulation, quality of life, and long-term outcomes in individuals with MS^[^5–7^]^.

Recent studies suggest that individuals with MS may be affected by metabolic abnormalities, including metabolic syndrome (MetS)^[^8^]^. Several factors could contribute to this association, including reduced physical activity related to neurological disability, intermittent or long-term corticosteroid exposure, disease-modifying therapies, diet, smoking, and other lifestyle or comorbidity patterns^[^9,10^]^. However, the extent to which these factors independently explain MetS risk in MS remains uncertain because many available studies are cross-sectional and adjust incompletely for potential confounders^[^11–14^]^.

From a clinical perspective, the coexistence of MS and MetS poses significant challenges. MetS may contribute to faster disability progression, increased fatigue, cognitive dysfunction, and diminished response to immunomodulatory treatments^[^15,16^]^. Moreover, cardiovascular disease has emerged as an important contributor to morbidity and mortality in MS^[^17^]^, underscoring the need for a comprehensive assessment of metabolic risk in routine clinical care. Despite these concerns, estimates of MetS prevalence in MS populations remain inconsistent across studies, with reported rates varying widely depending on study design, diagnostic criteria for MetS, demographic characteristics, geographic regions, and disease-related factors such as disease duration and treatment exposure^[^18^]^. A clear understanding of the burden of MetS in MS is essential to inform clinical decision-making and preventive strategies.

To our knowledge, this is the first systematic review and meta-analysis assessing the pooled prevalence of MetS in patients with MS. By synthesizing the existing body of evidence and examining sources of heterogeneity, this study aims to deepen our understanding of metabolic comorbidities in MS. Furthermore, the findings will support the development of integrated, multidisciplinary approaches to patient care, ultimately enhancing clinical practices and improving health outcomes for individuals with MS.

## Methods

### Protocol and registration

This systematic review and meta-analysis were conducted in accordance with the Preferred Reporting Items for Systematic Reviews and Meta-Analyses (PRISMA) guidelines^[^19^]^. The study protocol was prospectively registered with the International Prospective Register of Systematic Reviews (PROSPERO; registration ID: CRD420251269605).

### Literature search strategy

A comprehensive and systematic literature search was conducted in PubMed/MEDLINE, Embase, Web of Science, and Scopus from inception to 29 October 2025. The search strategy combined controlled vocabulary (e.g., MeSH and Emtree terms) and free-text keywords related to MS and MetS. Key search terms included combinations of “Metabolic Syndrome,” “Metabolic Syndrome X,” “Multiple Sclerosis,” and “Disseminated Sclerosis.” Search terms were combined using Boolean operators and adapted to the syntax of each database. No trial or study registries were searched. In addition, the reference lists of included studies and relevant review articles were manually screened to identify further eligible studies. No restrictions were applied regarding geographic location. Only studies published in English were included. The full search strategy is presented in Supplemental Digital Content Table S1, available at: https://links.lww.com/MS9/B416.

## S*tudy selection and eligibility criteria*

All records obtained from the literature search were entered into reference management software, and any duplicate entries were eliminated. Two reviewers independently screened the titles and abstracts to determine which studies might be eligible for inclusion. The full texts of articles deemed relevant were subsequently retrieved and assessed for inclusion.

HIGHLIGHTS

- Metabolic syndrome(MetS) affects one-quarter of patients with multiple sclerosis (MS; 25%; 95% CI, 20–31%). The prevalence reached 58% in patients with a body mass index (BMI) ≥ 30.
- Patients with expanded disability status scale (EDSS) ≥ 4 had a higher prevalence of MetS (51%) than those with EDSS < 4 (28%).
- MS patients with MetS were significantly older (Hedges’ *g* = 0.66). No differences were observed in age at diagnosis or disease duration.

Studies were deemed eligible if they involved individuals diagnosed with MS based on recognized diagnostic criteria, reported the prevalence of MetS in patients with MS, or provided adequate data to calculate prevalence estimates. Additionally, the studies needed to use a clearly defined and widely accepted diagnostic criteria for MetS and follow an observational study design, such as cross-sectional, cohort, or case–control. Studies were excluded if they failed to present relevant or extractable data regarding MetS in populations with MS, were interventional studies lacking usable baseline prevalence data, or fell into categories such as case reports, small case series, narrative reviews, editorials, letters, conference abstracts, or studies involving non-human subjects or experimental designs. In cases where several publications were based on the same or overlapping populations of patients with MS, the study with the most comprehensive or latest data was selected for inclusion. Disagreements concerning study eligibility were addressed through discussion, and if needed, a third reviewer was consulted.

## Q*uality assessment*

The methodological quality of the studies included in this review was assessed independently by two reviewers using the Joanna Briggs Institute Critical Appraisal Checklist designed for observational research. In cases in which the reviewers disagreed on the quality ratings, discussions were held to reach consensus, and a third reviewer was consulted when necessary.

## D*ata extraction*

Data were independently extracted by two reviewers using a standardized data extraction form. The following information was recorded for each included study: the first author, year of publication, and country or region; study design and setting; sample size and demographic characteristics; MS-related characteristics, such as disease duration, MS subtype, and MS diagnostic criteria; MetS diagnostic criteria; and the number of MS patients diagnosed with MetS and the reported prevalence. Any discrepancies in the data extraction process were resolved through discussion and, if necessary, with the involvement of a third reviewer to reach consensus.

## S*tatistical analyses*

All statistical analyses were performed using Stata software, version 19.5. For each study, the proportion of individuals with MS who were diagnosed with MetS was either extracted from the literature or calculated as necessary. Pooled prevalence estimates and 95% confidence intervals were computed using a random-effects model to account for variability among studies.

The degree of heterogeneity across studies was assessed using Cochran’s *Q* test and quantified with the *I*^2^ statistic. An *I*^2^ value greater than 50%, along with a *P*-value below 0.10 for the *Q* test, indicated substantial heterogeneity. When applicable, subgroup analyses were performed based on geographic region, definitions of MetS, diagnostic criteria for MetS used in the original studies, and study design to uncover potential sources of heterogeneity. Additionally, meta-regression analyses were conducted to further investigate heterogeneity at the study level.

To evaluate publication bias and the influence of smaller studies, we conducted visual inspections of funnel plots and applied Begg’s rank correlation test and Egger’s regression test; a *P*-value under 0.10 was regarded as indicative of possible publication bias. Galbraith plots were reviewed to identify any outliers. When data were available, we also examined the association between MetS and selected demographic and clinical factors in individuals with MS, where possible.

## Results

### Study selection

The systematic search identified 948 records from four databases: PubMed/MEDLINE (*n* = 95), Embase (*n* = 154), Web of Science (*n* = 216), and Scopus (*n* = 483). No registry records were searched or included. After the removal of 304 duplicate records, 644 studies remained for title and abstract screening. Of these, 606 records were excluded based on predefined eligibility criteria. The full texts of 38 articles were subsequently assessed for eligibility; of these, 31 were excluded. Ultimately, seven studies met the inclusion criteria and were included in the quantitative synthesis. The study selection process is summarized in Figure. 1.

**Figure 1.**
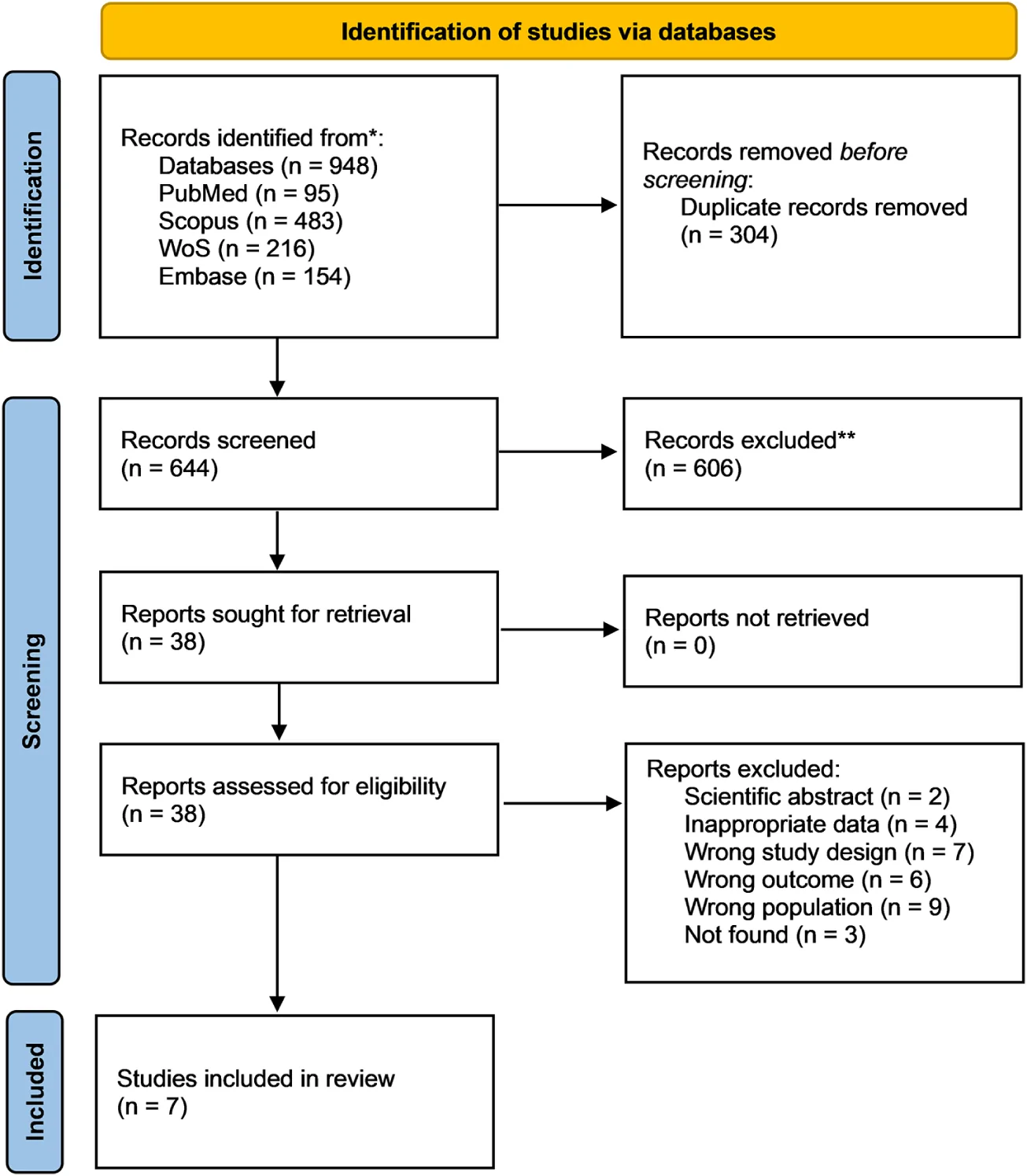
Study selection process.

### Study characteristics

The characteristics of the included studies are presented in Table 1. Of the seven included studies, five were cross-sectional, one was a cohort study, and one was a case–control study. In terms of geographical distribution, two studies were conducted in Asia, three in Europe, and two in Africa.

**Table 1:** Baseline characteristics of included studies.

No.	Authors	Study design	Continents	Sample size	Age, years	Male sample	Patients with MetS (*n*)	MS criteria	MetS criteria	EDSS	Disease duration	MS diagnosed age	BMI (kg/m^2^)	HDL (mg/dL)	LDL (mg/dL)	WC (cm)	Triglycerides (mg/dL)	Cholesterol (mg/dL)	SBP (mmHg)
1	Barzegar *et al* 2025^[^4^]^	Cross-sectional	Asia	225	40.46 ± 9.39	52	35	NR	AHA/NHLBI	2 (1.0–4.0)	11.25 ± 6.17	29.23 ± 8.47	NR	NR	NR	NR	NR	NR	NR
2	Fahmi *et al* 2020^[^20^]^	Cross-sectional	Africa	60	31.4 ± 8.41	18	22	McDonald	NCEP/ATP III	2.81 ± 1.9	4.35 ± 3.89	27.05 ± 8.01	NR	44.33 ± 13.49	122.09 ± 33.85	NR	140.87 ± 42.33	193.89 ± 36.87	120.75 ± 19.48
3	Gauthier *et al* 2024^[^21^]^	Cross-sectional	Europe	84	45.5 [38.0–57.0]	28	IDF: 23/NCEP ATP III: 23	McDonald	NCEP/ATP III	2.5 [1.5–3.5]	16.5 [8.8–22.5]	28 [21.0–37.3]	23.1 [20.7–25.7]	58 [46–70]	NR	88 [79.0–96.3]	87 [65–120]	NR	120 [110–128]
4	Pinhas–Hamiel *et al* 2015^[^18^]^	Cohort	Asia	130	55.8 ± 6	36	39	McDonald	NCEP/ATP III	5.5 ± 1	18.2 ± 10.1	NR	NR	NR	NR	NR	NR	NR	NR
5	Sircas–Mainar *et al* 2017^[^22^]^	Case-control	Europe	222	NR	79	69	ICPC-2/ ICD-9	NCEP/ATP III	3.2 ± 2	13.4 ± 9.5	NR	26 ± 4.5	59.5 ± 17.4	121 ± 37.4	NR	108.7 ± 49.9	199.6 ± 39.1	127.6 ± 15.5
6	Soliman *et al* 2020^[^23^]^	Cross-sectional	Africa	50	34.1 ± 6.7	16	11	McDonald	NCEP/ATP III	2.9 ± 1.4	7.3 ± 5.1	34.1 ± 6.8	NR	49.6 ± 6.4	117.3 ± 48.5	83.8 ± 14	103.9 ± 53.2	NR	121.2 ± 17.5
7	Suliga *et al* 2022^[^24^]^	Cross-sectional	Europe	115	42.6 ± 11.6	28	23	NR	IDF	NR	NR	31.7 ± 9.7	NR	62.7 ± 12.4	NR	85.7 ± 13.7	96.6 ± 44.5	NR	121.1 ± 13.4

BMI, body mass index; HDL, high-density lipoprotein; LDL, low-density lipoprotein; WC, waist circumference; SBP, systolic blood pressure; MetS, metabolic syndrome; MS, multiple sclerosis; EDSS, Expanded Disability Status Scale; SLE, systemic lupus erythematosus; NR, not reported; AHA/NHLBI, American Heart Association/National Heart, Lung, and Blood Institute; NCEP/ATP III, National Cholesterol Education Program Adult Treatment Panel III; IDF, International Diabetes Federation; McD, McDonald criteria; ICD-9, International Classification of Diseases, Ninth Revision; ICPCs, International Classification of Primary Care, Second Edition.

For diagnosis of MS, four studies used the McDonald criteria, and one study applied the International Classification of Diseases, Ninth Revision (ICD-9) or International Classification of Primary Care, Second Edition (ICPC-2) criteria. In two studies, the MS diagnostic criteria were not reported clearly.

MetS was assessed using established definitions, including the National Cholesterol Education Program Adult Treatment Panel III (NCEP/ATP III), the International Diabetes Federation (IDF) definition, and the American Heart Association/National Heart, Lung, and Blood Institute (AHA/NHLBI) criteria. Five studies reported MetS prevalence using NCEP/ATP III criteria, two using the IDF definition, and one using AHA/NHLBI criteria. One study reported MetS prevalence separately according to both the IDF definition and NCEP/ATP III criteria, and these were treated as separate effect sizes in the subgroup analysis. The quality of the studies is presented in Supplemental Digital Content Figure S1, available at: https://links.lww.com/MS9/B416.

### Prevalence of MetS among MS patients

The pooled prevalence of MetS among patients with MS was 0.25 (95% CI: 0.20–0.31), with substantial heterogeneity (*I*^2^ = 71.87%) (Fig. 2A). In leave-one-out sensitivity analyses, the pooled prevalence remained similar after omitting each study sequentially, ranging from 0.24 to 0.28 (Fig. 2B). The 95% prediction interval for the pooled prevalence was 0.099–0.451, indicating that the expected prevalence may vary substantially across future MS populations. The Galbraith plot identified Barzegar *et al*^[^4^]^ as an outlier study (Fig. 2C). Because this study reported a relatively low MetS prevalence and used the AHA/NHLBI definition, it likely contributed to between-study heterogeneity; however, the leave-one-out analysis showed that removing any single study, including this outlier, did not materially alter the overall conclusion.

**Figure 2.**
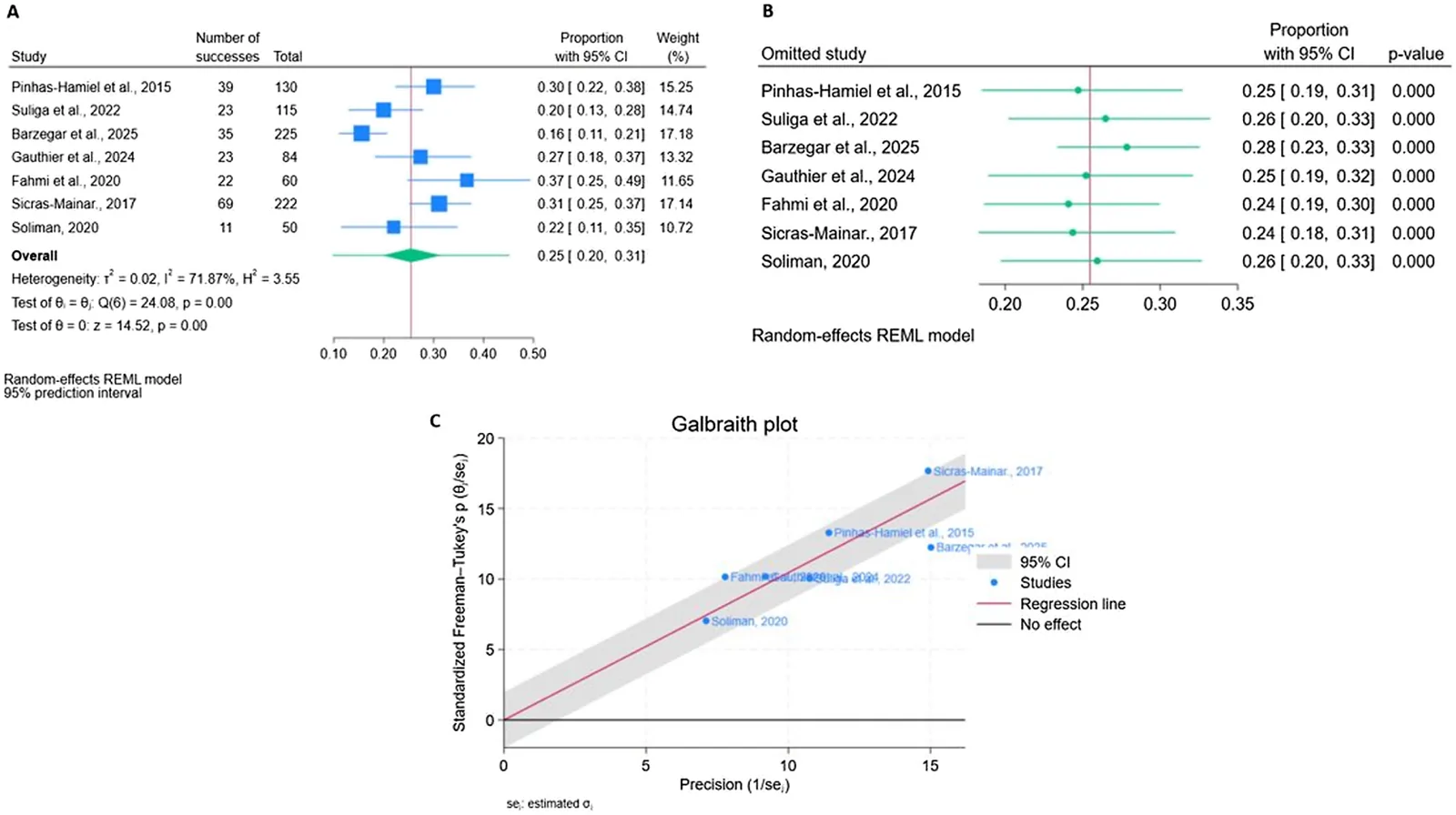
Meta-analysis of the prevalence of metabolic syndrome among patients with multiple sclerosis. (A) Forest plot. (B) Sensitivity analysis. (C) Galbraith plot.

### Subgroup analysis by diagnostic criteria

### MS criteria

In subgroup analyses based on MS diagnostic criteria, four studies that used the McDonald criteria reported a pooled MetS prevalence of 0.29 (95% CI, 0.24–0.34), with no observed heterogeneity (*I*^2^ = 0.00%). One study using ICPC-2/ICD-9 criteria reported a MetS prevalence of 0.31 (95% CI, 0.25–0.37). These subgroup findings should be interpreted cautiously because some categories included only a single study.

### MetS criteria

In subgroup analysis according to MetS diagnostic criteria, five studies reporting MetS based on NCEP/ATP III criteria yielded a pooled prevalence of 0.30 (95% CI, 0.26–0.34), with no observed heterogeneity (*I*^2^ = 0.00%). Two studies using the IDF definition reported a pooled prevalence of 0.23 (95% CI, 0.17–0.31), with moderate heterogeneity (*I*^2^ = 31.98%). One study applying the AHA/NHLBI criteria reported a MetS prevalence of 0.16 (95% CI, 0.11–0.21) (Table 2). Because some diagnostic-criteria subgroups included only one or two studies, these estimates are best viewed as descriptive rather than definitive evidence of differences between criteria.

**Table 2:** Results of subgroup analyses of metabolic syndrome prevalence among patients with multiple sclerosis.

Variable	Number of effect size	Prevalence	95% CI	*I*^2^ (%)	Tau^2^	*H*^2^
MS
MetS	7	0.25	(0.20–0.31)			
MS diagnostic criteria
McDonald	4	0.29	(0.24–0.34)	0.00	0.00	1.00
ICPC2/ICD-9	1	0.31	(0.25–0.37)	–	0.00	–
MetS diagnostic criteria
IDF	2	0.23	(0.17–0.31)	31.98	0.005	1.47
AHA/NHLBI	1	0.16	(0.11–0.21)	–	0.00	–
NCEP/ATP III	5	0.30	(0.26–0.34)	0.00	0.00	1.00
Continent
Asia	1	0.22	(0.10–0.38)	–	0.00	–
Europe	2	0.26	(0.20–0.33)	0.00	0.00	1.00
Africa	2	0.29	(0.16–0.44)	63.60	0.032	2.75
Type of study
Case-control	1	0.22	(0.12–0.35)	–	0.00	–
Cohort	1	0.31	(0.25–0.37)	–	0.00	–
Cross-sectional	3	0.31	(0.25–0.36)	0.00	0.00	1.00
BMI
BMI < 25	2	0.11	(0.02–0.26)	77.39	0.053	4.42
25 ≤ BMI < 30	2	0.31	(0.21–0.42)	0.00	0.00	1.00
BMI ≥ 30	2	0.58	(0.42–0.73)	0.00	0.00	1.00
EDSS
EDSS < 4	2	0.28	(0.21–0.34)	0.00	0.00	1.00
EDSS ≥ 4	2	0.51	(0.19–0.83)	84.83	0.199	6.52

MS, multiple sclerosis; MetS, metabolic syndrome; BMI, body mass index; EDSS, Expanded Disability Score Scale.

### Subgroup analysis based on BMI categories

Subgroup analysis according to BMI categories showed that in patients with BMI < 25, the pooled prevalence of MetS was 0.11 (95% CI, 0.02–0.26), with substantial heterogeneity (*I*^2^ = 77.39%). Among patients with 25 ≤ BMI < 30, the pooled prevalence was 0.31 (95% CI, 0.21–0.42), with no heterogeneity observed (*I*^2^ = 0.00%). In patients with BMI ≥ 30, the pooled prevalence of MetS was 0.58 (95% CI, 0.42–0.73), with no heterogeneity observed (*I*^2^ = 0.00%). Detailed results are presented in Table 2.

### Subgroup analysis based on EDSS

In a subgroup analysis based on EDSS, studies reporting EDSS < 4 showed a pooled MetS prevalence of 0.28 (95% CI, 0.21–0.34), with no observed heterogeneity (*I*^2^ = 0.00%). In contrast, studies reporting EDSS ≥ 4 demonstrated a pooled prevalence of 0.51 (95% CI, 0.19–0.83), with substantial heterogeneity (*I*^2^ = 84.83%) (Table 2). This finding suggests that higher disability may coexist with a higher metabolic burden, but the estimate is imprecise and should not be interpreted as evidence that disability-related inactivity causes MetS.

### Subgroup analysis by geographical region

Subgroup analysis by geographical region indicated that the pooled prevalence of MetS was 0.22 (95% CI, 0.10–0.38) in studies from Asia, 0.26 (95% CI, 0.20–0.33) in Europe, and 0.29 (95% CI, 0.16–0.44) in Africa. Heterogeneity was not observed in studies from Europe (*I*^2^ = 0.00%), whereas substantial heterogeneity was present among studies from Africa (*I*^2^ = 63.60%) (Table 2).

### Risk factors

### Age

Among patients with MS, those with MetS were older than those without MetS, with a pooled Hedges’ *g* of 0.66 (95% CI, 0.30–1.02; *P* < 0.01) and moderate heterogeneity (*I*^2^ = 45.63%) (Fig. 3A). In leave-one-out sensitivity analyses, the pooled estimate remained generally consistent after omitting each study sequentially (Fig. 3B). Given the small number of included studies, funnel plot assessment was not performed; however, there was no evidence of small-study effects based on Egger’s test (*P* = 0.28) and Begg’s test (*P* = 0.30).

**Figure 3.**
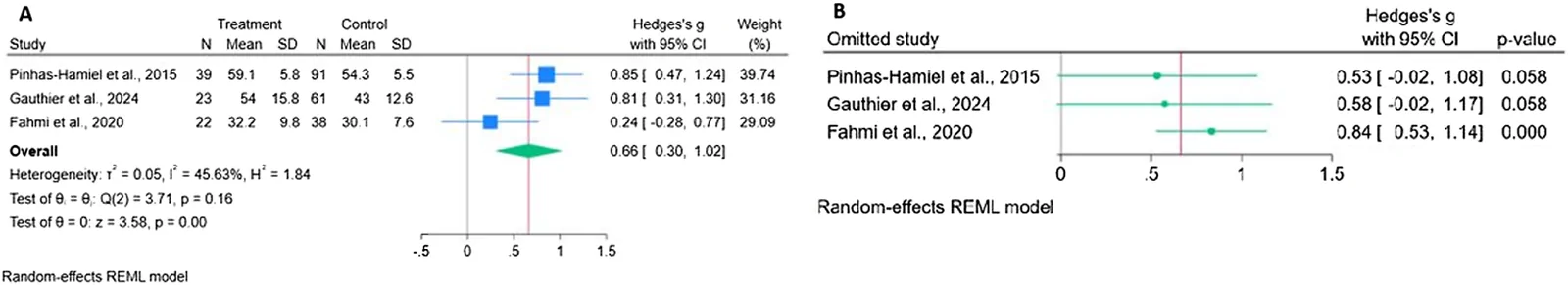
Forest plot comparing age between patients with multiple sclerosis with and without metabolic syndrome. (A) Forest plot. (B) Sensitivity analysis.

### Age at diagnosis of MS

For age at diagnosis of MS, the pooled analysis showed no clear difference between patients with and without MetS, with a Hedges’ *g* of 0.15 (95% CI, −0.21–0.50, *P* = 0.42) and no observed heterogeneity (*I*^2^ = 0.00%). The pooled estimate was not statistically significant. Owing to the limited number of included studies, funnel plot assessment was not performed (Fig. 4).

**Figure 4.**
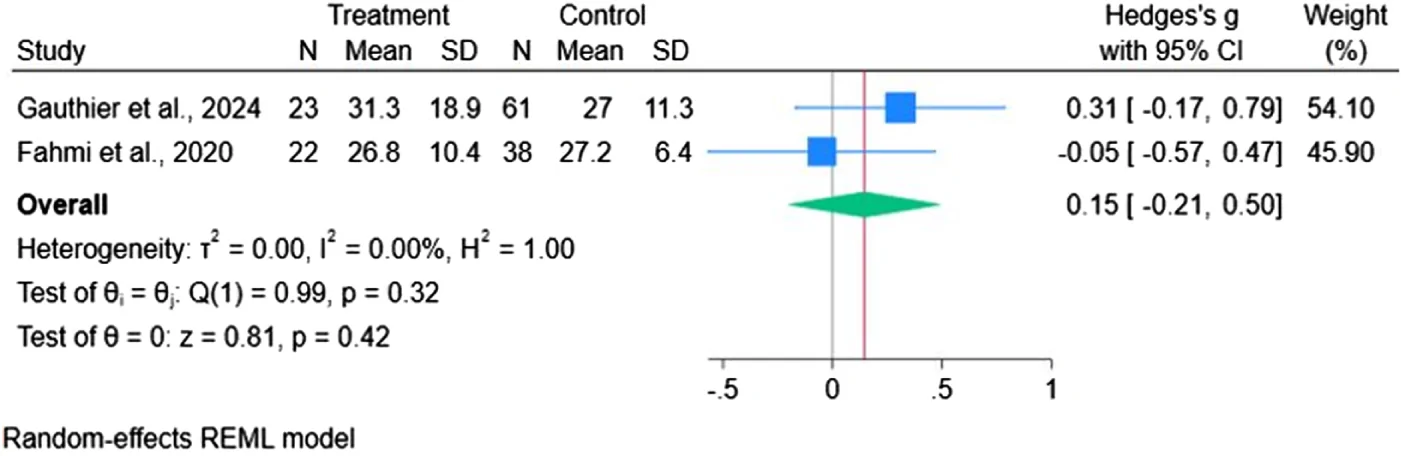
Forest plot comparing age at diagnosis between patients with multiple sclerosis, with and without metabolic syndrome.

### Disease duration

For disease duration, the pooled analysis showed no clear difference between patients with MS, with and without MetS, with a Hedges’ *g* of 0.06 (95% CI, −0.25–0.36; *P* = 0.72) and low heterogeneity (*I*^2^ = 26.77%) (Fig. 5A). Leave-one-out sensitivity analysis demonstrated that the pooled estimate remained stable after sequential omission of individual studies (Fig. 5B). Because of the small number of included studies, funnel plot assessment was not performed; however, no evidence of small-study effects was observed based on both Egger’s test (*P* = 0.33) and Begg’s test (*P* = 0.30).

**Figure 5.**
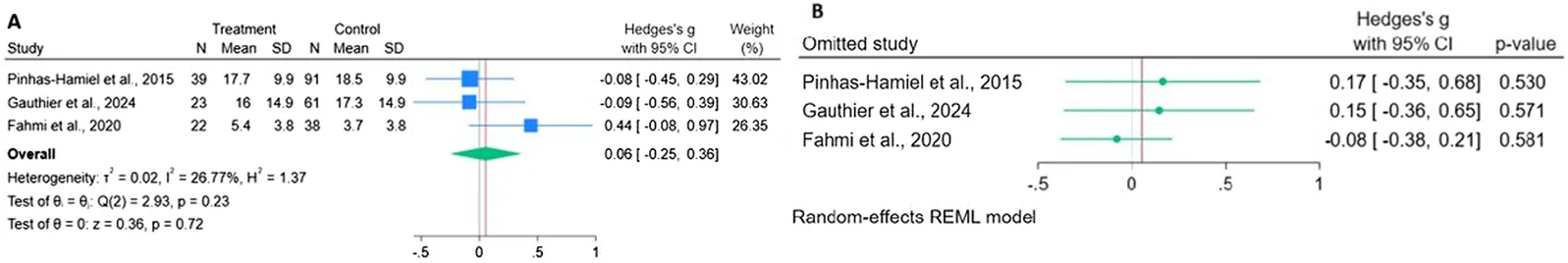
Forest plot comparing disease duration between patients with multiple sclerosis with and without metabolic syndrome. (A) Forest plot. (B) Sensitivity analysis.

### Results of meta-regression

Meta-regression analyses were conducted to explore potential sources of heterogeneity in MetS prevalence. None of the examined covariates, including HDL (*β* = −0.011, *P* = 0.284), LDL (*β* = 0.060, *P* = 0.105), WC (*β* = 0.036, *P* = 0.376), TG (β = 0.006, *P* = 0.092), SBP (*β* = 0.014, *P* = 0.56), DBP (β = −0.025, *P* = 0.184), FBS (*β* = 0.005, *P* = 0.179), smoking (*β* = −0.002, *P* = 0.760), and EDSS (*β* = 0.064, *P* = 0.304), showed a statistically significant association. Residual heterogeneity varied across models, with residual *I*^2^ values ranging from 0.00% to 84.35%, as shown in Table 3 and Figure 6. These analyses should be interpreted cautiously because the number of included studies was small, and several important confounders, including physical activity, corticosteroid exposure, disease-modifying therapy, diet, smoking, disability severity, and comorbidities, were missing or inconsistently reported across studies.

**Figure 6.**
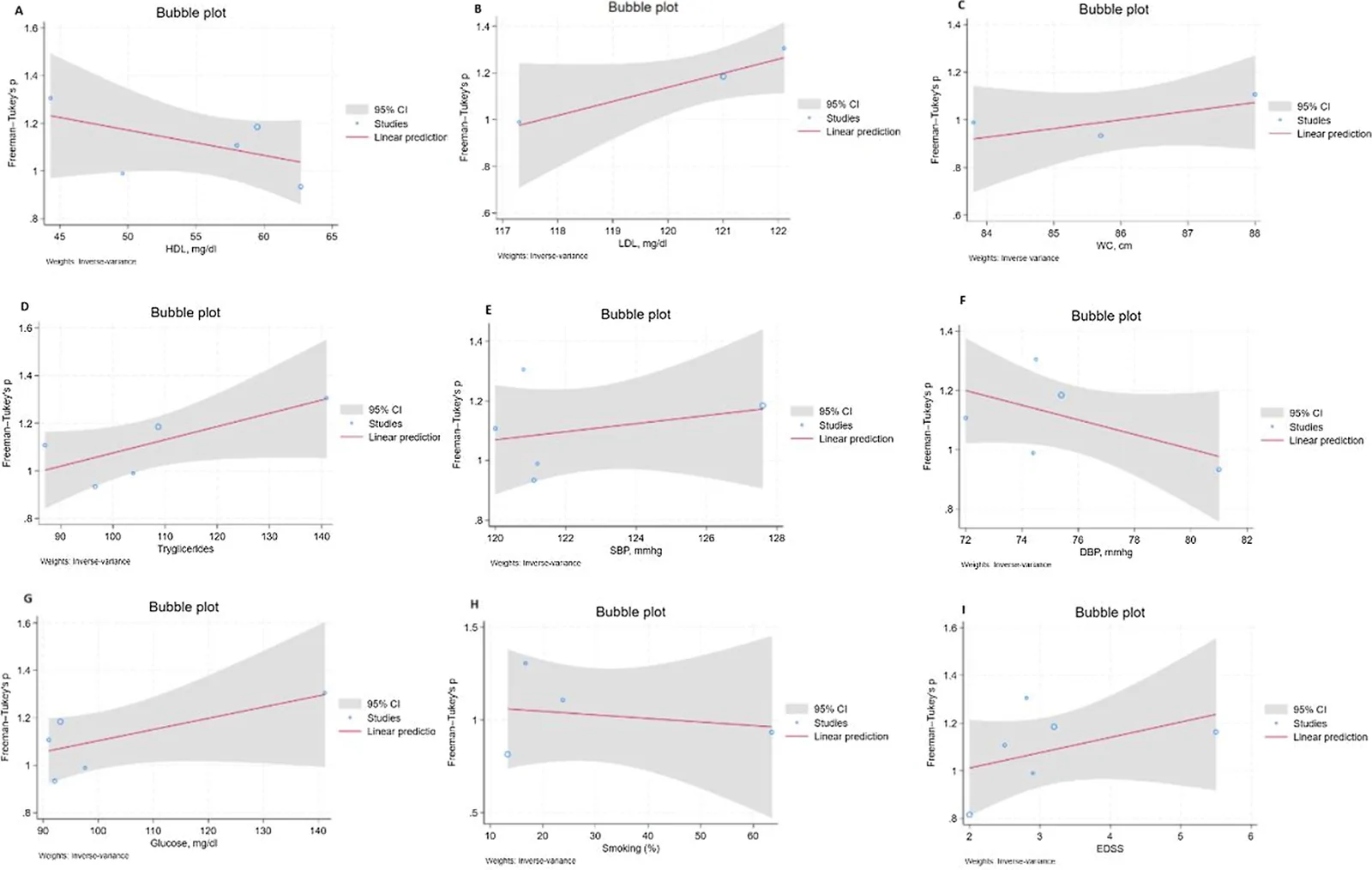
Results of the meta-regression analysis.

**Table 3:** Results of meta-regression.

Variable	Coefficient (*β*)	*P-*value	Residual Tau^2^	Residual *I*^2^	Residual *H*^2^	*R*^2^
HDL	−0.011	0.284	0.009	49.35	1.97	4.18
LDL	0.060	0.105	0.00	0.01	1.00	0.00
WC	0.036	0.376	0.00	0.00	1.00	8.90
TG	0.006	0.092	0.004	28.20	1.39	62.03
SBP	0.014	0.56	0.014	51.85	2.08	0.00
DBP	−0.025	0.184	0.006	34.79	1.53	42.78
FBS	0.005	0.179	0.008	46.04	1.85	18.13
Smoking	−0.002	0.760	0.056	84.35	6.39	0.00
EDSS	0.064	0.304	0.022	71.05	3.45	6.55

HDL, high density lipoprotein; LDL, low density lipoprotein; WC, waist circumference; TG, triglyceride; SBP, systolic blood pressure; DBP, diastolic blood pressure; FBS, fasting blood sugar; EDSS, Expanded Disability Score Scale.

## Discussion

In this systematic review and meta-analysis, we evaluated the prevalence of MetS in patients with MS. Across seven observational studies, MetS affected approximately one in four patients with MS. However, the evidence base was small, and the pooled estimate should be interpreted with caution because substantial heterogeneity was present. Pooled analyses of patient characteristics suggested that patients with MetS were older than those without MetS, whereas age at MS onset and disease duration showed no clear differences. Subgroup analyses suggested a higher prevalence among patients with elevated BMI and higher EDSS scores, but these analyses were based on a few studies and should be considered exploratory. Prior evidence came from cross-sectional and cohort studies with variable prevalence estimates, and differences in sample size and diagnostic criteria contributed to heterogeneity^[^18,22,25^]^. To our knowledge, this is the first meta-analysis to quantitatively synthesize data on MetS specifically in MS.

The substantial heterogeneity observed across studies warrants cautious interpretation. Variability in MetS prevalence may reflect differences in MetS definitions, MS diagnostic criteria, study design, geographic setting, age distribution, disability burden, BMI distribution, treatment exposure, and lifestyle factors. Barzegar *et al*^[^4^]^ was identified as an outlier in the Galbraith plot and reported a relatively low MetS prevalence using AHA/NHLBI criteria. Nevertheless, leave-one-out sensitivity analyses showed that the pooled prevalence remained within a narrow range after sequential exclusion of individual studies, indicating that the main conclusion was not driven by this single study. Subgroup analyses suggested a graded pattern in which MetS prevalence was higher among patients with elevated BMI. A similar descriptive pattern was observed for higher EDSS scores, although the EDSS ≥ 4 subgroup showed substantial heterogeneity and very wide confidence intervals. These findings are biologically plausible because reduced mobility and lifestyle factors may contribute to cardiometabolic risk in some patients with MS^[^26^]^; however, causality cannot be inferred from the available data. Most included studies were cross-sectional and did not consistently report or adjust for physical activity, diet, corticosteroid exposure, disease-modifying therapy, smoking, or comorbidities^[^27^]^. These findings underscore the importance of considering disability-related lifestyle factors when interpreting the distribution of MetS in this population.

Several mechanisms could plausibly link MS and MetS, but the included prevalence studies cannot establish these pathways directly. MetS is associated with low-grade systemic inflammation, endothelial dysfunction, dysregulated adipokine signaling, insulin resistance, and oxidative stress, all of which may overlap with processes relevant to neuroinflammation and vascular health^[^28–30^]^. MetS is characterized by chronic low-grade systemic inflammation, with elevated circulating proinflammatory cytokines such as TNF-α, IL-6, IL-1β, and high-sensitivity CRP^[^31^]^. This systemic inflammatory milieu can potentiate the activation of autoreactive T and B cells, thereby amplifying central nervous system inflammation and promoting demyelinating processes characteristic of MS^[^2,32^]^. Concurrent endothelial dysfunction, including reduced nitric oxide bioavailability and impaired vascular homeostasis, increases blood–brain barrier permeability, facilitating the transmigration of autoreactive immune cells into the CNS^[^33^]^. Dysregulated adipokine signaling, marked by elevated leptin and decreased adiponectin, further skews immune responses toward a proinflammatory Th1/Th17 phenotype while impairing regulatory T-cell function, reinforcing immune-mediated tissue damage. In addition, metabolic-syndrome-related insulin resistance and dyslipidemia increase oxidative stress and generate reactive oxygen species, exacerbating endothelial injury and compromising cerebral microvascular integrity^[^34^]^. These perturbations disrupt perfusion in vulnerable CNS regions and enhance recruitment of immune cells to sites of inflammation^[^35^]^. Collectively, these interrelated inflammatory, vascular, and metabolic disturbances create a self-perpetuating cycle in which systemic metabolic dysfunction promotes CNS inflammation, immune cell infiltration, and demyelination, thereby accelerating disease progression in susceptible individuals^[^36,37^]^. These mechanisms provide biological plausibility for an association between metabolic and neurological health, but they should be interpreted as contextual explanations rather than findings proven by the present meta-analysis.

Patients with MS and MetS tended to be older than those without MetS, consistent with the age-related accumulation of cardiometabolic risk. In contrast, no significant differences were observed in age at MS diagnosis or disease duration between individuals with and without MetS. These findings suggest that age may be an important correlate of MetS in MS, but the available evidence does not allow conclusions about whether MS duration or disease course directly influences MetS development^[^38^]^.

Meta-regression did not identify statistically significant study-level predictors of MetS prevalence. This finding should not be interpreted as evidence that these factors are unimportant. Rather, the analyses were underpowered because of the small number of studies and incomplete reporting of key variables, including physical activity, corticosteroid exposure, disease-modifying therapy, diet, smoking, disability severity, and comorbidities. From a clinical standpoint, the high prevalence of MetS in patients with MS underscores the need for systematic metabolic screening, particularly in those with elevated BMI or greater neurological disability^[^39,40^]^. Early identification of at-risk individuals can enable targeted, multidisciplinary interventions – including lifestyle modification, nutritional counseling, and cardiovascular risk management – consistent with current recommendations for metabolic risk assessment in MS care^[^41^]^.

Taken together, the available evidence suggests that MetS is a relevant comorbidity in MS, but the direction and mechanisms of association remain uncertain. Future prospective studies should collect standardized data on MS phenotype, EDSS, BMI, physical activity, diet, smoking, corticosteroid exposure, disease-modifying therapy, and other comorbidities to clarify whether metabolic risk is a cause, consequence, or correlate of disability and disease course in MS.

## Limitations

Several limitations should be considered. First, only seven observational studies were included, which reduced the precision of the overall estimate and limited the reliability of subgroup analyses and meta-regression. Second, substantial between-study heterogeneity was observed in the main pooled prevalence estimate and in the EDSS ≥ 4 subgroup. Third, many subgroup analyses included only one or two studies and should, therefore, be interpreted as exploratory. Fourth, variability in diagnostic criteria for MS and MetS across studies may have influenced prevalence estimates. Fifth, important confounders such as physical activity, corticosteroid exposure, disease-modifying therapy, diet, smoking, disability severity, and comorbidities were missing or inconsistently reported. Sixth, the predominance of cross-sectional designs limits inference on temporality or causality, and the manuscript cannot determine whether disability-related inactivity drives metabolic risk. Finally, the geographic coverage was limited, which may restrict generalizability to other settings and populations.

## Conclusion

This systematic review and meta-analysis suggest that MetS is present in approximately one-quarter of patients with MS, although the estimate is limited by the small number of available studies and substantial heterogeneity. Subgroup analyses suggested a higher MetS prevalence in patients with elevated BMI and higher EDSS scores, but these findings should be considered exploratory because several subgroups contained only one or two studies. Pooled analyses showed that patients with MetS were older than those without MetS, whereas age at MS diagnosis and disease duration did not clearly differ. Further prospective studies using standardized MS and MetS definitions and consistent reporting of lifestyle, treatment, disability, and comorbidity variables are needed to refine prevalence estimates and clarify potential determinants of metabolic risk in MS.

## Data Availability

Data can be provided on a resealable request from corresponding author. The datasets used and/or analyzed during the current study can be provided from the corresponding author upon reasonable request.
